# Geographic Access to Pediatric Cancer Care in the US

**DOI:** 10.1001/jamanetworkopen.2022.51524

**Published:** 2023-01-19

**Authors:** Xiaohui Liu, Mark N. Fluchel, Anne C. Kirchhoff, Haojie Zhu, Tracy Onega

**Affiliations:** 1 Department of Population Health Sciences, Huntsman Cancer Institute, University of Utah, Salt Lake City; 2 Division of Pediatric Hematology-Oncology, Seattle Children's Hospital, Seattle, Washington; 3 Department of Pediatrics, University of Washington School of Medicine, Seattle; 4 Department of Pediatrics, School of Medicine, Cancer Control and Population Sciences, Huntsman Cancer Institute, University of Utah, Salt Lake City

## Abstract

**Importance:**

Although access to pediatric cancer care has implications for use of such care and patient outcomes, little is known about the geographic accessibility of pediatric cancer care and how it may vary by population characteristics across the continental US.

**Objective:**

To estimate the travel time to pediatric cancer care settings in the continental US, identify potential disparities among subgroups of children and adolescents and young adults (AYAs), and identify areas needing improved access to pediatric cancer care.

**Design, Setting, and Participants:**

This cross-sectional study collected data from August 1 to December 1, 2021. Pediatric oncologists’ service locations in 2021 served as the pediatric cancer care settings, data for which were scraped from 2 websites containing information about health professionals. Demographic characteristics for younger children and AYAs aged 0 to 21 years were obtained from the 2015 to 2019 American Community Survey 5-year estimates. Data were analyzed from January 1 to April 31, 2022.

**Main Outcomes and Measures:**

The main outcome was the travel time from geographic centroids of zip code tabulation areas to the nearest pediatric oncologist. The median (IQR) travel times for each demographic subgroup were estimated. Per capita pediatric oncologist supply was calculated by dividing the total number of pediatric oncologists for each state or US Census division by its population.

**Results:**

Of the 90 498 890 children and AYAs included in the study, 63.6% were estimated to travel less than 30 minutes and 19.7% to travel between 30 and 60 minutes (for a total of 83.3%) to the nearest pediatric oncologist. Median (IQR) travel times were longest for the American Indian or Alaska Native pediatric population (46 [16-104] minutes) and residents of rural areas (95 [68-135] minutes), areas with high deprivation levels (36 [13-72] minutes), and the South (24 [13-47] minutes) and Midwest (22 [11-51] minutes) compared with the general population of children and AYAs. The pediatric oncologist supply was lowest in Wyoming (0 oncologists per 100 000 pediatric population) and highest in Washington, DC (53.3 oncologists per 100 000 pediatric population). Pediatric oncologist supply across Census divisions was lowest in the Mountain division (3.3 oncologists per 100 000 pediatric population) and highest in the New England division (8.1 oncologists per 100 000 pediatric population).

**Conclusions and Relevance:**

Results of this study showed that most children and AYAs in the continental US had adequate access to pediatric cancer care, although disparities existed among racial and ethnic groups and residents in rural areas, areas with high deprivation levels, and some Southern and Midwestern states. Reducing these disparities may require innovative approaches, such as expanding the capabilities of local facilities and creating partnerships with adult oncology centers and primary care physicians.

## Key Points

**Question:**

What is the estimated travel time to access pediatric cancer care, and how does travel time vary by population characteristics across the continental US?

**Findings:**

In this cross-sectional study of more than 90 million children and adolescents and young adults (AYAs), 83.3% would travel less than 1 hour to visit the nearest pediatric oncologist. Median travel times were longest for the American Indian or Alaska Native pediatric population and those living in rural areas, areas with high deprivation index, and the South and Midwest.

**Meaning:**

The findings that disparities exist among racial and ethnic groups and residents in rural areas, areas with high deprivation levels, and some Southern and Midwestern states suggest the need for innovative approaches to reduce these disparities.

## Teaser

This cross-sectional study assesses the travel time required to reach US pediatric cancer facilities and clinicians as well as the factors that may impede this access within the 48 contiguous states.

## Introduction

Childhood cancer typically requires specialized treatment by a team of pediatric oncology practitioners who understand the unique needs of children and young adults with cancer.^1^ Those teams are usually found in highly specialized care centers, such as academic centers, children’s hospitals, and cancer centers, which are often located in urban areas. This locality creates challenges for families residing in rural or remote areas.^1^ Many factors can pose challenges in accessing pediatric cancer care, such as distance, travel time, region, and oncology service supply.^1,2^ Specifically, extended travel distance and travel time may impede the timeliness, frequency, and duration of care access, leading to lower use of care services.^3^ Similarly, extended travel times are associated with a greater financial burden on patients and families.^1^

Per capita oncologist supply is another important measure of geographic access to cancer care, as it gauges the extent to which the cancer care workforce can meet the demand for pediatric oncology services. To our knowledge, no studies have estimated the population-level per capita pediatric oncologist supply and the geographic variation in that supply; such a study is a fundamental step in ascertaining the adequacy of pediatric oncology services in the US.

Social determinants of health, such as race and ethnicity, health insurance status, and rurality, likely are associated with geographic access to pediatric cancer care.^4,5,6^ Improvements in pediatric cancer treatment have played a role in substantial increases in 5-year relative survival rates from 58% in the mid-1970s to 85% in 2021.^7^ However, evidence suggests that increased survival has benefited some groups more than others, especially those with cancers that are more amenable to treatment.^8^ A review by Beltrami et al^9^ demonstrated that racial, ethnic, and socioeconomic disparities in pediatric cancer outcomes exist across disease types. Health insurance status also affects care-seeking behaviors, as evidenced in a study that reported that uninsured childhood cancer survivors had a high risk of forgoing care regardless of sociodemographic background.^10^ Rural residents were found to have later-stage cancer at diagnosis and a lower survival rate compared with those living in metropolitan areas.^11^

The association of socioeconomic status (SES) with cancer outcomes, including pediatric cancer, has also been recognized.^4,12,13^ Given that individual-level SES data are hard to obtain, area-level measures of SES, such as Area Deprivation Index (ADI), have been widely and reliably used as the proxy variable.^5^ Previous studies documented that socioeconomic factors played a substantial role in pediatric cancer outcomes.^5,9,14,15^ In particular, deprivation was associated with poor overall survival in pediatric patients with acute lymphoblastic leukemia.^5^ Another study found that deprivation, as measured by ADI, was associated with poor survival for all childhood cancers combined in a cohort of pediatric patients with cancer.^16^ Household material hardship for families has also been shown to have increasing impact while their child is undergoing pediatric chemotherapy.^17^

Although earlier reports have examined travel burden for pediatric patients with cancer,^1^ no study has estimated the travel time aspect of the geographic accessibility of pediatric cancer care settings. The goal of this cross-sectional study was to estimate the travel time to pediatric cancer care settings in the continental US. Furthermore, to identify potential disparities among subgroups of children and adolescents and young adults (AYAs), we compared the travel time for these subgroups based on demographic and geographic characteristics. In addition, to identify areas needing improved access to pediatric cancer care, we calculated the per capita pediatric oncologist supply on state and Census division levels.

## Methods

### Data Collection

Pediatric oncologist data, including name, practicing address, and National Provider Identifier (NPI), were collected from doximity.com and NPIDB.org^18^ using web scraping. Doximity.com has a profile for most US medical professionals, which includes basic information gathered from public sources and NPI data, and serves as a social network site for health professionals.^19^ NPIDB.org is based on the information that clinicians filled out while applying for an NPI number. We scraped data on 4368 unique pediatric oncologists, and their service addresses were geocoded to latitudes and longitudes using Geocoding API (Google Developers).^20^ This cross-sectional study was a secondary analysis of publicly available population data and thus, according to the Common Rule, was exempt from institutional review board review and the informed consent requirement. We followed the Strengthening the Reporting of Observational Studies in Epidemiology (STROBE) reporting guideline.

To ensure the accuracy of the web-scraped data, we compared the number of pediatric oncologists in the data with that in external sources, such as physician listings (DocSpot and *U.S. News & World Report*) and hospital websites. We randomly selected 1 state from each of the 9 US Census Bureau divisions as the spot-checking site. The number of pediatric oncologists in web-scraped data correlated well with the number in external sources, with a correlation coefficient of 0.99, suggesting that the methods we used reliably captured the actual number of pediatric oncologists in the continental US (eAppendix in Supplement 1).

Pediatric oncologists were mapped in ArcGIS 10.7 (Esri) using coordinates, and later a 5-digit zip code tabulation area (ZCTA) TIGER/Line Shapefile for 2019 (US Census Bureau) was included to identify 1059 ZCTAs with at least 1 pediatric oncologist.^21^ The centroids of those ZCTAs served as the 2021 locations of pediatric oncologists in calculations of geographic access to pediatric care services. The population centroid of each ZCTA was identified as the geographic centroid of each ZCTA.

### Demographic Data

Demographic characteristics in each ZCTA were obtained from the 2015 to 2019 American Community Survey (ACS) 5-year estimates (US Census Bureau).^22^ Based on the age limit of pediatrics set by the American Academy of Pediatrics, we chose the upper age limit of 21 years for the study population.^23^ For the race and ethnicity variable, we chose American Indian or Alaska Native, Asian, Black or African American, Hispanic or Latino, and White. For race and ethnicity categories only, we used the age range of 0 to 19 years because the US Census Bureau summarized race and ethnicity data using 19 years as a breakdown age. Other categories were still based on the 0 to 21 years age range of the American Academy of Pediatrics.^22^ We also dichotomized age categories into younger children (aged 0-14 years) and AYAs (aged 15-21 years), because AYAs may use either pediatric or adult cancer care centers for specific treatments, while younger children typically use only pediatric cancer care centers. Health insurance status was dichotomized into insured and uninsured for those aged 0 to 18 years; these data were also derived from the ACS estimates.^22^

### Rurality and Area Deprivation Index

To attribute the rurality of residence to individual zip codes, we used the Rural-Urban Commuting Area (RUCA) classification system, version 3.1 (US Department of Agriculture), which was based on the 2006 to 2010 ACS 5-year estimates^24^ and commuting flow. Rural status was determined by linking the zip code to its corresponding RUCA 4-tier classifications: urban, large town, small town, and rural.^24^ The 48 continental states and Washington, DC, were divided into 4 regions based on the US Census Bureau regions and divisions.^25^

The ADI is a geography-based social determinant of health that brings together geography from the 2019 TIGER/Line Shapefile and data from the 2015 to 2019 ACS 5-year estimates.^26,27,28^ The ADI uses individual socioeconomic variables, including annual income, occupation, housing ownership, occupation status, and educational level, to calculate the ADI level. We used national ADI percentile data, which were ranked 1 to 100, with a higher rank indicating a higher deprivation level. The average zip code–level ADI was estimated from the 9-digit zip code–level ADI within a given 5-digit zip code area. Then, we classified the national ADI score into tertiles: low deprivation (score of 0-33), median deprivation (score of 34-66), and high deprivation (score of 66-100).

### Geographic Access

Travel times to pediatric oncologists were calculated using the shortest travel time from each ZCTA centroid to the nearest ZCTA with at least 1 pediatric oncologist. We used network analysis in ArcGIS 10.7 to find the fastest path by comparing travel time in minutes between each origin-destination pair while considering travel distances and speed limits within a 5-hour driving distance. The origin-destination pair with the shortest travel time was selected for each origin (ZCTA centroid), and the travel time was used as the shortest time to a pediatric oncologist for the population in that ZCTA. Travel times to other ZCTAs with at least 1 pediatric oncologist were summarized to derive the median, maximum, 25th percentile, and 75th percentile travel time within the 5-hour driving distance. We used 6 travel time categories: less than 30 minutes, 30 to less than 60 minutes, 1 to less than 2 hours, 2 to less than 3 hours, 3 to less than 4 hours, and 4 hours or more. Some origin-destination pairs (3.2%) had the same origin and destination; thus, the travel time was zero and was underestimated. We calculated the per capita pediatric oncologist supply for each state by dividing the total number of pediatric oncologists by the entire population of children and AYAs, and then multiplying by 100 000. We also calculated the per capita pediatric oncologist supply for 9 Census divisions.

### Statistical Analysis

Zip codes to ZCTA crosswalk files were used to link data that were indexed with zip codes (RUCA and ADI) and with ZCTA (pediatric demographic data and travel times) using the open-source Python library pandas.^29^ Shortest travel times were mapped in ArcPro 2.9 for the 48 contiguous states and Washington, DC. Median travel times by race and ethnicity, age category, health insurance status, rurality, ADI level, and region were calculated using Stata, version 17 (StataCorp LLC). As this analysis was applied to the entire US population in the study site, we did not conduct tests of statistical significance.

Data were collected from August 1 to December 1, 2021. Data analysis was performed from January 1 to April 31, 2022. 

## Results

The total number of children and AYAs in this study was 90 498 890. This population comprised children and AYAs with the following race and ethnicity: 0.9% American Indian or Alaska Native, 4.2% Asian, 12.6% Black or African American, 22.3% Hispanic or Latino, and 60.0% White (Table).

**Table.** Median Travel Time and Census-Level Demographic Characteristics for Children and AYAs (N = 90 498 890)

Characteristic	No. (%), millions	Median travel time (IQR), min
Total population: aged 0-21 y	90.5 (100)	20 (10-42)
Race and ethnicity for individuals aged 0-19 y^a^		
American Indian or Alaska Native	0.78 (0.9)	46 (16-104)
Asian	3.87 (4.2)	12 (7-20)
Black or African American	11.53 (12.6)	14 (8-31)
Hispanic or Latino	20.33 (22.3)	15 (8-31)
White	54.78 (60.0)	24 (12-49)
Age categories		
Younger children: 0-14 y	60.62 (66.9)	20 (10-43)
AYAs: 15-21 y	29.87 (33.1)	21 (10-44)
Health insurance status for individuals aged 0-18 y^b^		
Insured	73.27 (94.9)	20 (10-43)
Uninsured	3.91 (5.1)	22 (11-49)
Rurality		
Urban	75.8 (83.8)	16 (9-30)
Large town	8.13 (9.0)	67 (46-103)
Small town	3.83 (4.2)	82 (57-116)
Rural	2.34 (2.6)	95 (68-135)
ADI level		
Low deprivation	26.8 (29.6)	12 (7-22)
Median deprivation	37.21 (41.1)	22 (12-41)
High deprivation	26.57 (29.4)	36 (13-72)
Region		
Northeast	14.71 (16.3)	11 (5-26)
Midwest	19.26 (21.3)	22 (11-51)
South	35.15 (38.8)	24 (13-47)
West	21.36 (23.6)	17 (10-39)

Abbreviations: ADI, Area Deprivation Index; AYA, adolescent and young adult.

^a^ Population size: 91 294 908, because Hispanic or Latino was not an exclusive category.

^b^ Population size: 77 198 398.

**Figure 1.**
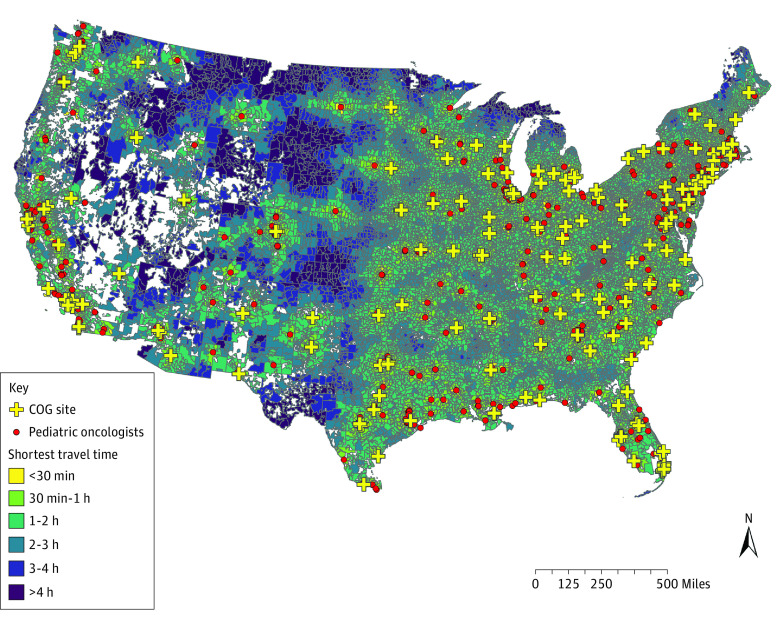
Shortest Travel Time From Population Centroids of US ZIP Code Tabulation Areas to the Nearest Pediatric Oncologist Patients within the yellow zip code tabulation areas need to travel less than 30 minutes to visit a pediatric oncologist. Travel times increase as the map color darkens, and the dark blue areas indicate more than 4 hours of travel time. COG indicates Children’s Oncology Group.

Travel time calculation included 32 658 ZCTAs from the 48 continental states and Washington, DC. Of these ZCTAs, 1059 (3.2%) contained at least 1 of the 4368 pediatric oncologists. Among the children and AYAs, 63.6% were estimated to travel less than 30 minutes to the nearest pediatric oncologist, 19.7% to travel between 30 and 60 minutes, 12.4% to travel between 1 and 2 hours, 2.9% to travel between 2 and 3 hours, 0.8% to travel between 3 and 4 hours, and 0.5% to travel for 4 hours or more. The median (IQR) travel time was 20 (10-42) minutes. To illustrate how travel times varied among ZCTAs across the continental US, Figure 1 shows the shortest 6 travel time categories.

Median (IQR) travel times for American Indian or Alaska Native children and AYAs, residents in rural areas, areas with high deprivation level, and the Southern region were the longest compared with the general population of children and AYAs. Asian children and AYAs had the shortest median (IQR) travel time to pediatric oncologists (12 [7-20] minutes) compared with the American Indian or Alaska Native pediatric population who had the longest travel time (46 [16-104] minutes). Black or African American and Hispanic or Latino patients had a similar median (IQR) travel time (14 [8-31] and 15 [8-31] minutes, respectively). White children and AYAs had a longer median (IQR) travel time at 24 (12-49) minutes (Table).

Median (IQR) travel times for younger children (aged 0-14 years) and AYAs (aged 15-21 years) were similar (20 [10-43] and 21 [10-44] minutes, respectively). For people with or without health insurance, median (IQR) travel times were also similar (20 [10-43] and 22 [11-49] minutes, respectively) (Table).

Travel times to the nearest pediatric oncologist increased as rurality increased (Table). The median (IQR) travel time was 16 (9-30) minutes for 83.8% of children and AYAs in urban areas, 67 (46-103) minutes for 9.0% in large towns, 82 (57-116) minutes for 4.2% in small towns, and 95 (68-135) minutes for 2.6% in rural areas. Travel times for ADI levels revealed a similar pattern for rurality levels (Table). The median (IQR) travel time was 12 (7-22) minutes for 29.6% of the pediatric population in low deprivation areas, 22 (12-41) minutes for 41.1% in median deprivation areas, and 36 (13-72) minutes for 29.4% in high deprivation areas. Regional differences in travel time were also observed. The median (IQR) travel time was 11 (5-26) minutes in the Northeast, 17 (10-39) minutes in the West, 22 (11-51) minutes in the Midwest, and 24 (13-47) minutes in the South.

**Figure 2.**
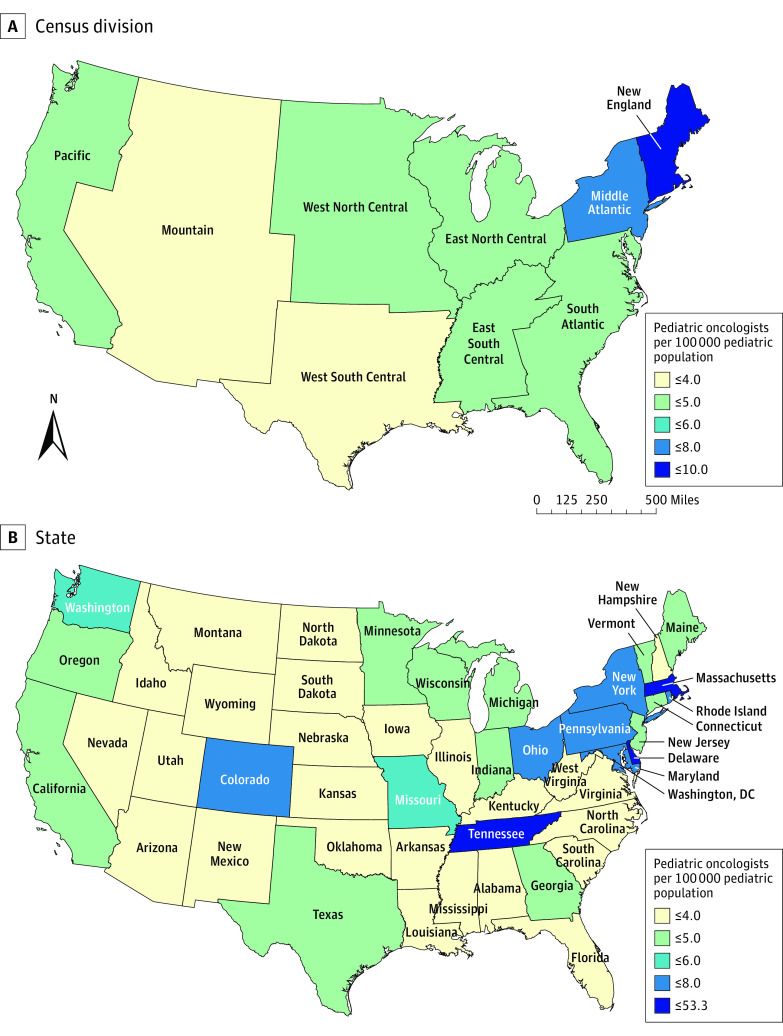
Per Capita Pediatric Oncologist Supply by Census Division and State

The pediatric oncologist supply by Census division showed notable differences, with the Mountain division having the lowest per capita supply (3.3 oncologists per 100 000 pediatric population) (Figure 2A) and New England having the highest per capita supply (8.1 oncologists per 100 000 pediatric population). Other divisions in the central part of the US (range, 3.7-4.9 oncologists per 100 000 pediatric population) had a much lower per capita supply than the Middle Atlantic (6.7 oncologists per 100 000 pediatric population) and New England divisions (8.1 oncologists per 100 000 pediatric population). When analyzed at the state level, stark differences in supply emerged (Figure 2B). Most states in the Northeast had a high per capita supply, such as Maryland (7.8 oncologists per 100 000 pediatric population) and Pennsylvania (7.4 oncologists per 100 000 pediatric population) (Figure 2B). States in the Midwest had the lowest supply, such as Wyoming (0 oncologists per 100 000 pediatric population), Idaho (1.3 oncologists per 100 000 pediatric population), and Montana (1.4 oncologists per 100 000 pediatric population). The supply was highest in Washington, DC, at 53.3 oncologists per 100 000 pediatric population. States in the West had more per capita supply than states in the Midwest and the South.

## Discussion

Geographic access to pediatric oncology care varied widely in the continental US, with notable disparities among racial and ethnic groups, urban and rural areas, ADI levels, and regions. In particular, American Indian or Alaska Native patients and those living in rural areas, areas with high deprivation levels, and the Midwest and South had the highest travel times to the nearest pediatric oncological care. Analysis of the pediatric oncologist supply revealed that states in the Northeast and West had more per capita pediatric oncologists than other regions.

The variations in geographic access to pediatric oncologists across racial and ethnic groups, urban and rural areas, and regions are comparable to variations found in an earlier study of adult cancer care settings.^2^ That study reported that American Indian or Alaska Native individuals, nonurban residents, and people in the South bore relatively greater burden when traveling to any specialized cancer care settings than their counterparts,^2^ which concurs with the findings in this study. While the factors associated with the travel burden are similar for adults and children and AYAs, the actual travel time is much greater for children and AYAs than for adults because pediatric cancer care is more often delivered at children’s hospitals in large academic centers in urban areas.^2^

We also found that decreased geographic access to pediatric cancer care was associated with the ADI.^28^ The ADI has been validated for various health outcomes and disease domains at the neighborhood level.^5,17^ As a geography-based social determinant of health, increased ADI was associated with disadvantages in physical access, further supporting previous findings that social determinants also have implications for the health outcomes of the pediatric population.^5,17^

While complex social determinants of health likely underlie these differences, reducing disparities in geographic access may require innovative approaches. Given the small number of patients with cancer that would fully use such a service in remote locations or through the Indian Health Service, establishing more Children’s Oncology Group sites in rural areas might not be feasible. However, expanding the capabilities of local facilities to include services, such as central line access, antibiotic administration, blood transfusion, and laboratory evaluation, may alleviate the travel burden for patients. Pediatric patients with cancer, especially older adolescents, who are not treated in Children’s Oncology Group sites or comprehensive cancer care centers often have inferior outcomes.^30^ Furthermore, creating partnerships with adult oncology centers, advanced practitioners, and primary care physicians may allow for the administration of uncomplicated outpatient chemotherapy drug, such as vincristine, thereby keeping a substantial proportion of care visits closer to home. In addition, expanding telehealth for those visits when laboratory work, physical examination, or chemotherapy is not needed may address some of the geographic access issues identified.

The difference in pediatric oncologist supply between states in the Northeast vs the Midwest showed a severe regional disparity that warrants further investigation to help alleviate the burden of care for families of pediatric patients with cancer. It is unclear whether this disparity has adverse implications for the diagnosis and treatment of pediatric cancer. Northeastern states had the highest pediatric cancer rates between 2003 and 2014, including New Hampshire (205.5 incident cases per 1 million population); Washington, DC (194 incident cases per 1 million population); and New Jersey (192 incident cases per 1 million population).^31^ Also notable was that the Northeastern states had the highest relative survival rates compared with other regions, as evidenced in a survival study of 185 312 pediatric patients with cancer during 2001 to 2015.^32^ While we can draw no direct causal association from the results of the present study, access to highly subspecialized pediatric oncology centers is critical to the appropriate diagnosis and treatment of childhood cancer.^32^

### Limitations

This study has several limitations. The travel time analysis was conducted on population-level estimates but not on a sample of pediatric patients with cancer. Therefore, the study provides a general and simplified simulation of the travel time on a national scale. This estimated travel time may serve as empirical evidence for further analysis that incorporates more comprehensive factors (eg, pediatric cancer types) and more accurate spatial factors (eg, urban congestion and public transport). However, this study did not account for spatially heterogeneous rates of pediatric cancers, which may limit the usefulness of the findings. Furthermore, the analysis excluded Alaska and Hawaii because travel in those states is often not road based; the study would be more accurate and appropriate in those states if other travel modes (eg, by train and air) were incorporated.

We demonstrated the reliability of the pediatric oncologist data that we scraped from NPIDB.org and doximity.com. Yet, limitations exist in the web-scraped data due to differences in data collection and update frequency between the 2 websites. Another limitation of the web-scraped data is the completeness of the pediatric oncologists’ practicing locations, as some clinicians may see patients in remote sites other than their primary locations. Similarly, we could not account for the possibility that advanced practitioners provided care in more remote areas. Therefore, the omission of remote care sites may have led to an overestimation of travel distances and travel times. Moreover, the greater number of pediatric oncologists in the Northeast and West may be somewhat skewed by the inclusion of those who focused on research and did limited clinical work. Future research needs to examine clinical efforts to improve the accuracy of this analysis.

## Conclusion

In this cross-sectional study of the travel time aspect of the geographic accessibility of pediatric cancer care, most children and AYAs in the continental US were estimated to have adequate access, although disparities existed among racial and ethnic groups as well as residents in rural areas, areas with high deprivation levels, and some Southern and Midwestern states. Reducing disparities in geographic access may require innovative approaches, such as expanding the capabilities of local facilities to include pediatric cancer–related services and creating partnerships with adult oncology centers and primary care physicians. States in the South and Midwest need more pediatric oncologists to ensure that patient needs are met.

## Notes

Supplement 1. **eAppendix.** Spot-Checking Number of Oncologists per State

Supplement 2.
Data Sharing Statement

## References

[1] Fluchel MN, Kirchhoff AC, Bodson J, . Geography and the burden of care in pediatric cancers. Pediatr Blood Cancer. 2014;61(11):1918-1924. doi:10.1002/pbc.25170 25131518 PMC4749153

[2] Onega T, Duell EJ, Shi X, Wang D, Demidenko E, Goodman D. Geographic access to cancer care in the U.S. Cancer. 2008;112(4):909-918. doi:10.1002/cncr.23229 18189295

[3] Wan N, Zhan FB, Zou B, Chow E. A relative spatial access assessment approach for analyzing potential spatial access to colorectal cancer services in Texas. Appl Geogr. 2012;32(2):291-299. doi:10.1016/j.apgeog.2011.05.001

[4] Adam M, Rueegg CS, Schmidlin K, ; Swiss Paediatric Oncology Group; Swiss National Cohort Study. Socioeconomic disparities in childhood cancer survival in Switzerland. Int J Cancer. 2016;138(12):2856-2866. doi:10.1002/ijc.30029 26840758

[5] Schraw JM, Peckham-Gregory EC, Rabin KR, Scheurer ME, Lupo PJ, Oluyomi A. Area deprivation is associated with poorer overall survival in children with acute lymphoblastic leukemia. Pediatr Blood Cancer. 2020;67(9):e28525. doi:10.1002/pbc.28525 32573920

[6] Kehm RD, Spector LG, Poynter JN, Vock DM, Altekruse SF, Osypuk TL. Does socioeconomic status account for racial and ethnic disparities in childhood cancer survival? Cancer. 2018;124(20):4090-4097. doi:10.1002/cncr.31560 30125340 PMC6234050

[7] American Cancer Society. Cancer Facts & Figures 2022. American Cancer Society; 2022.

[8] Delavar A, Barnes JM, Wang X, Johnson KJ. Associations between race/ethnicity and US childhood and adolescent cancer survival by treatment amenability. JAMA Pediatr. 2020;174(5):428-436. doi:10.1001/jamapediatrics.2019.6074 32091555 PMC7042928

[9] Beltrami A, Hilliard A, Green AL. Demographic and socioeconomic disparities in pediatric cancer in the United States: current knowledge, deepening understanding, and expanding intervention. Cancer Epidemiol. 2022;76:102082. doi:10.1016/j.canep.2021.102082 34923289

[10] Baedke JL, Lindsey LA, James AS, . Forgoing needed medical care among long-term survivors of childhood cancer: racial/ethnic-insurance disparities. J Cancer Surviv. 2022;16(3):677-687. doi:10.1007/s11764-021-01061-3 34046821 PMC8626536

[11] Darlington WS, Green AL. The role of geographic distance from a cancer center in survival and stage of AYA cancer diagnoses. Cancer. 2021;127(19):3508-3510. doi:10.1002/cncr.33666 34232508

[12] Friedrich P, Itriago E, Rodriguez-Galindo C, Ribeiro K. Racial and ethnic disparities in the incidence of pediatric extracranial embryonal tumors. J Natl Cancer Inst. 2017;109(10):djx050. doi:10.1093/jnci/djx050 29117360

[13] Bhatia S. Disparities in cancer outcomes: lessons learned from children with cancer. Pediatr Blood Cancer. 2011;56(6):994-1002. doi:10.1002/pbc.23078 21328525 PMC3369622

[14] Oates G, Rutland S, Juarez L, Friedman A, Schechter MS. The association of area deprivation and state child health with respiratory outcomes of pediatric patients with cystic fibrosis in the United States. Pediatr Pulmonol. 2021;56(5):883-890. doi:10.1002/ppul.25192 33258546 PMC8035176

[15] Singh GK, Lin CCC. Area deprivation and inequalities in health and health care outcomes. Ann Intern Med. 2019;171(2):131-132. doi:10.7326/M19-1510 31261382

[16] Bona K, London WB, Guo D, Frank DA, Wolfe J. Trajectory of material hardship and income poverty in families of children undergoing chemotherapy: a prospective cohort study. Pediatr Blood Cancer. 2016;63(1):105-111. doi:10.1002/pbc.25762 26398865

[17] Zhao J, Han X, Zheng Z, . Racial/ethnic disparities in childhood cancer survival in the United States. Cancer Epidemiol Biomarkers Prev. 2021;30(11):2010-2017. doi:10.1158/1055-9965.EPI-21-0117 34593561

[18] NPIDB. Lookup NPI numbers from the NPI Registry. Updated December 7, 2022. Accessed December 7, 2022. https://npidb.org/

[19] Doximity. U.S. healthcare professionals’ network. Accessed February 10, 2022. https://www.doximity.com

[20] Google Developers. Geocoding API. Accessed June 1, 2022. https://developers.google.com/maps/documentation/geocoding/usage-and-billing

[21] General Services Administration. TIGER/Line Shapefile, 2019, 2010 nation, U.S., 2010 Census 5-Digit ZIP Code Tabulation Area (ZCTA5) National. Accessed February 11, 2022. https://catalog.data.gov/dataset/tiger-line-shapefile-2019-2010-nation-u-s-2010-census-5-digit-zip-code-tabulation-area-zcta5-na

[22] US Census Bureau. 2015-2019 ACS 5-year estimates. 2019. Accessed February 11, 2022. https://www.census.gov/programs-surveys/acs/technical-documentation/table-and-geography-changes/2019/5-year.html

[23] Hardin AP, Hackell JM; Committee on Practice and Ambulatory Medicine. Age limit of pediatrics. Pediatrics. 2017;140(3):e20172151. doi:10.1542/peds.2017-2151 28827380

[24] Hailu A, Wasserman C. Guidelines for using rural-urban classification systems for community health assessment. 2016. Accessed February 14, 2022. https://www.doh.wa.gov/Portals/1/Documents/1500/RUCAGuide.pdf

[25] US Census Bureau. 2010 Census regions and divisions of the United States. 2010. Accessed February 14, 2022. https://www.census.gov/geographies/reference-maps/2010/geo/2010-census-regions-and-divisions-of-the-united-states.html

[26] Mora J, Krepline AN, Aldakkak M, . Adjuvant therapy rates and overall survival in patients with localized pancreatic cancer from high Area Deprivation Index neighborhoods. Am J Surg. 2021;222(1):10-17. doi:10.1016/j.amjsurg.2020.12.001 33308823

[27] University of Wisconsin School of Medicine Public Health. 2019 Area Deprivation Index v3.0. 2019. Accessed September 3, 2021. https://www.neighborhoodatlas.medicine.wisc.edu/

[28] Kind AJH, Buckingham WR. Making neighborhood-disadvantage metrics accessible: the neighborhood atlas. N Engl J Med. 2018;378(26):2456-2458. doi:10.1056/NEJMp1802313 29949490 PMC6051533

[29] McKinney W. Data structures for statistical computing in Python. In: van der Walt S, Millman J, eds. Proceedings of the 9th Python in Science Conference. SciPy; 2010:56-61. doi:10.25080/Majora-92bf1922-00a

[30] Wolfson J, Sun CL, Wyatt L, Stock W, Bhatia S. Impact of treatment site on disparities in outcome among adolescent and young adults with Hodgkin lymphoma. Leukemia. 2017;31(6):1450-1453. doi:10.1038/leu.2017.66 28218238 PMC5462848

[31] Galvin G. Childhood cancer hits prosperous counties, big cities the hardest. *US News & World Report*. June 28, 2018. Accessed March 21, 2022. //https://www.usnews.com/news/healthiest-communities/articles/2018-06-28/cdc-childhood-cancer-rates-highest-in-prosperous-counties-big-cities

[32] Siegel DA, Richardson LC, Henley SJ, . Pediatric cancer mortality and survival in the United States, 2001-2016. Cancer. 2020;126(19):4379-4389. doi:10.1002/cncr.33080 32725630 PMC9539939

