# Supplementary material for: Geographic Access to Pediatric Cancer Care in the US
Source: JAMA Netw Open. 2023 Jan 19;6(1):e2251524. doi: 10.1001/jamanetworkopen.2022.51524 (PMC9856631; doi:10.1001/jamanetworkopen.2022.51524)
Supplement: Supplement 1. — eAppendix. Spot-Checking Number of Oncologists per State [file jamanetwopen-e2251524-s001.pdf]

Supplemental Online Content

Liu X, Fluchel MN, Kirchhoff AC, Zhu H, Onega T. Geographic access to pediatric cancer care in the US. *JAMA Netw Open*. 2023;6(1):e2251524. doi:10.1001/jamanetworkopen.2022.51524

eAppendix. Spot-Checking Number of Oncologists per State

This supplemental material has been provided by the authors to give readers additional information about their work.

© 2023 Liu X et al. *JAMA Network Open.*

eAppendix. Spot-Checking Number of Oncologists per State

Census Division	State	Number of pediatric oncologists in web-scraped data	Number of pediatric oncologists in external sources
Pacific	Oregon	45	41 (DocSpot)
Mountain	Utah	31	31 (DocSpot)
West North Central	Minnesota	71	88 (DocSpot)
West South Central	Oklahoma	29	28 (DocSpot)
East North Central	Ohio	207	202 (DocSpot)
East South Central	Tennessee	168	180 (DocSpot)
South Atlantic	North Carolina	108	118 (DocSpot)
Middle Atlantic	New York	376	331 (U.S. News & World Report)
New England	Vermot	7	7 (DocSpot)

© 2023 Liu X et al. *JAMA Network Open*.
