# Supplementary material for: Geographic Access to Pediatric Cancer Care in the US
Source: JAMA Netw Open. 2023 Jan 19;6(1):e2251524. doi: 10.1001/jamanetworkopen.2022.51524 (PMC9856631; doi:10.1001/jamanetworkopen.2022.51524)
Supplement: Supplement 2. — Data Sharing Statement [file jamanetwopen-e2251524-s002.pdf]

Data Sharing Statement

Liu. Geographic Access to Pediatric Cancer Care in the US. *JAMA Netw Open*. Published January 19, 2023. doi:10.1001/jamanetworkopen.2022.51524

Data
Data available: No
